# Crop yield sensitivity of global major agricultural countries to droughts and the projected changes in the future

**DOI:** 10.1016/j.scitotenv.2018.10.434

**Published:** 2019-03-01

**Authors:** Guoyong Leng, Jim Hall

**Affiliations:** Environmental Change Institute, University of Oxford, Oxford OX1 3QY, UK

**Keywords:** Agricultural production, Climate change, Risk, Drought

## Abstract

Understanding the potential drought impacts on agricultural production is critical for ensuring global food security. Instead of providing a deterministic estimate, this study investigates the likelihood of yield loss of wheat, maize, rice and soybeans in response to droughts of various intensities in the 10 largest producing countries. We use crop-country specific standardized precipitation index (SPI) and census yield data for 1961–2016 to build a probabilistic modeling framework for estimating yield loss risk under a moderate (−1.2 < SPI < −0.8), severe (−1.5 < SPI < −1.3), extreme (−1.9 < SPI < −1.6) and exceptional (SPI < −2.0) drought. Results show that there is >80% probability that wheat production will fall below its long-term average when experiencing an exceptional drought, especially in USA and Canada. As for maize, India shows the highest risk of yield reduction under droughts, while rice is the crop that is most vulnerable to droughts in Vietnam and Thailand. Risk of drought-driven soybean yield loss is the highest in USA, Russian and India. Yield loss risk tends to grow faster when experiencing a shift in drought severity from moderate to severe than that from extreme to the exceptional category, demonstrating the non-linear response of yield to the increase in drought severity. Sensitivity analysis shows that temperature plays an important role in determining drought impacts, through reducing or amplifying drought-driven yield loss risk. Compared to present conditions, an ensemble of 11 crop models simulated an increase in yield loss risk by 9%–12%, 5.6%–6.3%, 18.1%–19.4% and 15.1%–16.1 for wheat, maize, rice and soybeans by the end of 21st century, respectively, without considering the benefits of CO_2_ fertilization and adaptations. This study highlights the non-linear response of yield loss risk to the increase in drought severity. This implies that adaptations should be more targeted, considering not only the crop type and region but also the specific drought severity of interest.

## Highlights

• The probability of yield loss under droughts is estimated for four major crops.
• Yield loss risk grows non-linearly with increase in drought severity.
• Yield loss risk is projected to increase in the future by an ensemble of models.

## Graphical abstract

Crop yield loss risk (%) in the past and future when experiencing a moderate, extreme, severe and exceptional drought. Each dot represents the multi-model ensemble mean, with the grey error lines denoting the uncertainty range.

**Figure:**
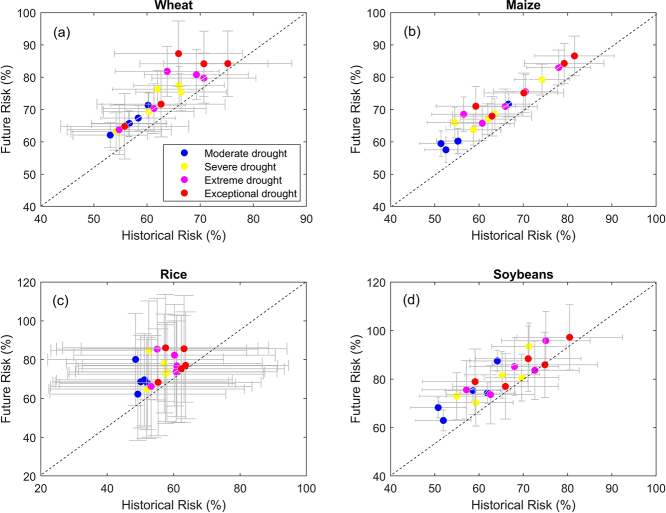

## 1 Introduction

Global food demand is expected to roughly double by 2050s (Godfray et al., 2010; Tilman et al., 2011). To meet growing food demand in the context of global warming requires enhanced understandings of the climatic factors influencing food production. Important in this regard is to examine how crop yield responds to climate variability and extremes. Typically, farmers would be more capable of adapting to the gradual changes in local mean climate conditions than extreme events, calling for the need of improved understanding of the impacts of climate extremes on agricultural production (Lesk et al., 2016; Zampieri et al., 2017). Drought, as an extreme weather phenomenon, is one of the major climatic constraints to crop yield (Lesk et al., 2016; Lobell et al., 2014; Matiu et al., 2017; Mishra and Cherkauer, 2010; Zipper et al., 2016). Under drought conditions, crops close their stomata to limit evaporative water loss, thus reducing carbon uptake by photosynthesis and decreasing yields. Globally, it is estimated that a cereal (maize, rice and wheat) loss of 1820 million Mg has been caused by droughts during the past four decades (Lesk et al., 2016).

Assessment of drought impact on agricultural production is challenging, especially given that drought itself is driven by complex interactions among precipitation, temperature, vapor pressure, and solar radiation. In addition, compound changes in other physical and agronomical factors influencing crop growth would complicate the assessment of yield response to droughts. For example, Leng, 2017a, Leng, 2017b showed that agricultural management such as irrigation can mitigate the negative impacts of water stress on maize yield, though the study was limited to US central High Plains. Recent process-based modeling studies also revealed large uncertainties in crop yield simulations arising from our incomplete knowledge of the physical and agronomical processes underlying crop growth and yield (Asseng et al., 2011; Elliott et al., 2015; Folberth et al., 2016; Rosenzweig et al., 2014). Thus, the incomplete understanding of the physical processes make it hard to derive certain estimates of drought impact on crop yield.

Previous studies have discussed the potential impact of drought on crop production in the United States (Lobell et al., 2014; Troy et al., 2015; Zipper et al., 2016), China (Shi and Tao, 2014; Yu et al., 2014), Australia (Madadgar et al., 2017), South Africa (Araujo et al., 2016), Republic of Moldova (Potopová et al., 2016), Czech Republic (Hlavinka et al., 2009), and the whole globe (Lesk et al., 2016; Matiu et al., 2017). Although these studies have provided valuable insights into the possible impacts of droughts, they are mainly based on deterministic approaches, reporting the overall yield fluctuation associated with drought. Recently, probabilistic methods have been adopted with the purpose of accounting for uncertainty from climate data (Tao et al., 2009; Tebaldi and Lobell, 2008). To date, probabilistic estimation of yield changes under a given drought of specific severity has not been conducted across global agricultural regions.

This study fills the gap by assessing the risk of crop yield loss under droughts in global major producing countries in a probabilistic manner. Specifically, we address the following scientific questions: 1) what are the possible changes in crop yield in response to an individual drought of specific severity? Understanding the distribution of possible outcomes of crop yield under a given drought can help decision-makers and insurers in selecting appropriate strategies based on the likelihood of different outcomes. 2) Does yield loss risk grow linearly with increase in drought severity? Examining yield sensitivity to droughts of various severity can better inform targeted adaptation and mitigations. 3) How will the likelihood of yield reduction change in the future?

## 2 Materials and methods

### 2.1 Crop yields and climate data

Country level census data on yields of the four largest commodity crops (i.e. wheat, maize, rice and soybeans) are collected from the Food and Agriculture Organization (http://faostat.fao.org) for the period 1961–2016. These four crops provide ~75% of all calories consumed by humans (Lobell et al., 2011). To investigate future changes in yield loss risk under droughts, we obtained crop yields simulated by 11 gridded crop models (Table 1) driven by five global climate models (Table 2) under RCP8.5 emission scenario from the Agricultural Modeling Inter-comparison and Improvement Project (AgMIP) (Rosenzweig et al., 2013) and the Inter-Sectoral Impact Model Inter-comparison Project (ISI-MIP) (Warszawski et al., 2014).

**Table 1:** Description of crop models used in this study.

Crop model	Model type	Key literature
CGMS-WOFOST	Spatially distributed site-based process model (based on WOFOST)	(de Wit and Van Diepen, 2008)
CLM-Crop	Global ecosystem model	(Drewniak et al., 2013)
GEPIC	Site-based process model (based on EPIC)	(Liu et al., 2007; Williams et al., 1983)
LPJ-GUESS	Global ecosystem model	(Lindeskog et al., 2013)
LPJmL	Global ecosystem model	(Waha et al., 2012)
pAPSIM	Site-based process model	(Keating et al., 2003)
PEGASUS	Global ecosystem model	(Deryng et al., 2016)
EPIC-IIASA	Site-based process model (based on EPIC)	(Izaurralde et al., 2006; Williams et al., 1983)
EPIC-Boku	Site-based process model (based on EPIC)	(Izaurralde et al., 2006; (Williams et al., 1983)
ORCHIDEE-crop	Global ecosystem model	(Wu et al., 2016)
pDSSAT	Site-based process model	(Jones et al., 2003)

**Table 2:** Descriptions of 5 GCMs used in this study.

Model name	Institute acronyms	Institute full name
GFDL-ESM2M	NOAA GFDL	NOAA Geophysical Fluid Dynamics Laboratory
HadGEM2-ES	MOHC (additional realizations by INPE)	Met Office Hadley Centre and Instituto Nacional de Pesquisas Espaciais
IPSL-CM5A-LR	IPSL	Institut Pierre-Simon Laplace
MIROC-ESM-CHEM	MIROC	Japan Agency for Marine-Earth Science and Technology, Atmosphere and Ocean Research Institute (The University of Tokyo), and National Institute for Environmental Studies
NorESM1-M	NCC	Norwegian Climate Centre

Gridded monthly climate data at 0.5^°^ × 0.5^°^ for the period 1961–2016 were obtained from the Climate Research Unit (Mitchell and Jones, 2005). Spatially weighted averages of gridded precipitation and temperature were computed for each crop and growing season based on weights defined by the crop area (Portmann et al., 2010), resulting in annual growing-season precipitation and temperature for each crop-country and crop-globe combination following (Lobell and Field, 2007). Growing seasons are determined from the crop calendar dataset developed by (Sacks et al., 2010). In this study, we focus on the top 10 producing countries that are highly relevant for global food market and security (Lobell, 2007; Matiu et al., 2017). The combined production of the top 10 productive countries accounts for 96%, 87%, 78% and 68% of global total production of soybeans, rice, maize and wheat, respectively. Yield variations in these major producing regions would therefore lead to significant anomalies at the global level, affecting global food market and security. Here, the FAO country-level yield data and observed climate are used to build the probabilistic model, based on which the historical sensitivity of crop yield to droughts is examined. To be consistent with observation-based analysis, the gridded crop model simulations are aggregated to country level based on which the projected changes in yield loss risk by the end of 21st century are investigated.

### 2.2 Probabilistic modeling of yield reduction risk under droughts

We develop a copula-based probabilistic model for estimating the possible responses of crop yields to a drought event of specific severity. To do this, we model the dependency between a drought index and crop yield using a copula function (Nelsen, 2007). Copulas enable modeling dependency between variables that do not follow the same distributions including non-normal distributions (Nelsen, 2007), thus avoiding assumptions about linearity or underlying probability distributions. In addition, copula is capable of proper treating the tails of distribution, which is critical for assessing extreme event like drought. In this study, five bivariate copula families, which are widely adopted in the literature, are used for fitting the joint probability distribution between a drought index (x) and crop yields (y) by

(1) $$F_{\mathrm{XY}}\left(X,Y\right)=C\left[F_{X}\left(X\right),F_{Y}\left(Y\right)\right]$$

where *F*_*X*_(*X*) and *F*_*Y*_(*Y*) are the marginal distributions of *x* and *y*, respectively. C is the cumulative distribution function (CDF) of copula. Details on these copula families and their mathematical descriptions can be found in Table 3. The conditional probability of crop yield dropping below a certain amount (*Y* < *y*) under a given drought event (*X* = *x*) can be expressed as follows:

(2) $$F_{Y|X}\left(Y<y\;\right|\;X=x)$$

**Table 3:** Copula families used in this study and the mathematical descriptions.

Name	Mathematical description	Parameter range	Reference
Gaussian	$\int_{-\infty }^{\emptyset^{-1}\left(u\right)}\int_{-\infty }^{\emptyset^{-1}\left(v\right)}\frac{1}{2\pi \sqrt{1-\theta^{2}}}\exp\left(\frac{2\mathrm{\theta xy}-x^{2}-y^{2}}{2\left(1-\theta^{2}\right)}\right)\mathrm{dxd}y^{b}$ *θ* : Linear correlation coefficient ∅ : Standard normal cumulative distribution function	*θ* ∈ [−1, 1]	(Renard and Lang, 2007)
t	$\int_{-\infty }^{t_{\theta_{2}}^{-1}\left(u\right)}\int_{-\infty }^{t_{\theta_{2}}^{-1}\left(v\right)}\frac{\Gamma \left(\frac{\theta_{2}+2}{2}\right)}{\Gamma \left(\frac{\theta_{2}}{2}\right)\pi \theta_{2}\sqrt{1-\theta_{1}^{2}}}{\left(1+\frac{x^{2}-2\theta_{1}\mathrm{xy}+y^{2}}{\theta^{2}}\right)}^{\frac{\theta_{2}+2}{2}}\mathrm{dxd}y^{c}$ *θ*_1_ : Linear correlation coefficient *t*_*θ*_2__ : Cumulative distribution function of *t* distribution with *θ*_2_ degree of freedom	*θ*_1_ ∈ [−1, 1]; *θ*_2_ ∈ [0, ∞]	(Demarta and McNeil, 2005)
Clayton	max(*u*^−*θ*^ + *v*^−*θ*^ − 1, 0)^−1/*θ*^ *θ* : Measure of dependency between *u* and *v*.	*θ* ∈ [−1, ∞]\0	(Clayton, 1978)
Frank	$-\frac{1}{\theta }\ln\left[1+\frac{\left(\exp\left(-\mathrm{\theta u}\right)-1\right)\left(\exp\left(-\mathrm{\theta v}\right)-1\right)}{\exp\left(-\theta \right)-1}\right]$ *θ* : Similar to Clayton copula	*θ* ∈ *ℝ*\0	(Li et al., 2013)
Gumbel	$\exp\left\{-{\left[{\left(-\ln\left(u\right)\right)}^{\theta }+{\left(-\ln\left(v\right)\right)}^{\theta }\right]}^{\frac{1}{\theta }}\right\}$ *θ* : Similar to Clayton copula	*θ* ∈ [−1, ∞]	(Zhang and Singh, 2006)

Based on the fitted copula, the conditional probability density distribution of *f*_*y*∣*x*_(*y*| *x*) is derived:

(3) $$f_{Y|X}\left(y\;\right|\;x)=c\left[F_{X}\left(X\right),F_{Y}\left(Y\right)\right]*f_{Y}\left(y\right)$$

where *c* is the copula, *f*_*Y*_(*y*) is the probability distribution function (PDF) of crop yield. The probability of crop yield dropping below a certain amount (i.e.,*F*_*Y*∣*X*_(*Y* < *y* | *X* = *x*)) can be estimated as the area under*f*_*Y*∣*X*_(*y*| *x*) for *Y* < *y*.

### 2.3 Uncertainty quantification

We use the Markov Chain Monte Carlo (MCMC) simulation technique within a Bayesian framework to infer the posterior distribution of copula parameters, based on which the uncertainty arising from our probabilistic model is quantified. The MCMC numerically solves the Bayes' equation to estimate the posterior distribution of copula parameter $p\left(\theta |\tilde{E}\right)$

(4) $$p\left(\theta |\tilde{E}\right)=\frac{p\left(\theta \right)p\left(\tilde{E}|\theta \right)}{p\left(\tilde{E}\right)}\propto p\left(\theta \right)p\left(\tilde{E}|\theta \right)$$

where *θ* is the copula parameter, $\tilde{E}$ is the empirical joint probability vector, *p*(*θ*) is the prior distribution of *θ*, while $p(\tilde{E}$) is the evidence. $p\left(\tilde{E}|\theta \right)$ is the likelihood function that is solved by assuming the error residual $\tilde{d_{i}}-d_{i}\left(\theta \right)$ between copula derived and empirical joint probability are uncorrelated.

(5) $$p\left(\tilde{E}|\theta \right)≅ℒ\left(\theta |\tilde{E}\right)=\prod_{i=1}^{n}\frac{1}{\sqrt{2\pi \tilde{\sigma }}}\exp\left\{-\frac{1}{2}{\tilde{\sigma }}^{-2}{\left[\tilde{d_{i}}-d_{i}\left(\theta \right)\right]}^{2}\right\}$$

where $\tilde{\sigma }$ is the standard deviation of measurement error estimated from MCMC simulations. Based on the posterior distribution of the parameters, the uncertainty range of our results can be derived.

### 2.4 Estimation of yield loss risk under droughts

Drought is multi-dimensional and can be grouped into meteorological, agricultural, hydrological and socio-economic droughts (Heim Jr, 2002; Huang et al., 2016; Leng et al., 2015; Mishra and Singh, 2010). In this study, we use the Standardized Precipitation Index (SPI) (McKee et al., 1993) to estimate the impacts of moisture supply originated from precipitation. A drought event is identified when the SPI value is below −0.8, following the U.S. Drought Monitor (http://droughtmonitor.unl.edu/). Yield changes under four drought categories (i.e. moderate, severe, extreme and exceptional droughts) are examined to quantify the sensitivity of yield response to the increase in drought severity (Table 4). Besides SPI, we use a drought index that includes the effects of temperature, given the critical role of temperature in crop growth and yield (Lobell et al., 2013; Lobell et al., 2014). We note that there are several indices that take into account the effects of temperature directly or indirectly, e.g., the standardized precipitation evapotranspiration index (*SPE*I) (Vicente-Serrano et al., 2010) and the Palmer Drought Severity Index (PDSI) (Palmer, 1965). Here, we select the *SPE*I for analysis, as it is an extension of SPI in the calculation procedures. The potential evapotranspiration (PET) is estimated based on the Thortnthwaite equation (Thornthwaite, 1948), instead of using other complex equations requiring more inputs. Comparing the results based on SPI and *SPE*I allows for examination on the compounding effects of temperature in modulating drought impacts.

**Table 4:** Classification of droughts following the U.S. Drought Monitor (http://droughtmonitor.unl.edu/).

Category	Range of SPI
Moderate drought	−1.2~−0.8
Severe drought	−1.5~−1.3
Extreme drought	−1.9~−1.6
Exceptional drought	≤−2.0

We first remove the linear temporal trend of crop yield using the least squares method to minimize the effects of slowly changing factors (e.g. agricultural management) (Hlavinka et al., 2009; Lobell et al., 2011). The five bi-variable copulas are then fitted to the de-trended crop yield and growing season SPI for each crop-country combination. We select the copula that has the highest statistically significant maximum likelihood as the best one (Sadegh et al., 2017), based on which the probability of yield loss (i.e. yield dropping below its long-term mean) under a given drought of specific severity is calculated. The statistical significance is estimated according to the two-tailed Student's *t*-test. We obtained similar results when using Bayesian Information Criterion (BIC) (Schwarz, 1978) and Akaike Information Criteria (AIC) (Akaike, 1981) as the evaluation metric.

The above analysis is repeated based on simulated crop yields by crop models driven by global climate models for the historical period (1961–2016) and future period (2071–2100) to explore future changes in yield loss risk by the end of 21st century. We note that several reference periods have been used in climate change impact assessment, such as 1980–2010 (Schewe et al., 2014), 1971–2000 (Haddeland et al., 2014) and 1986–2005 (Lissner and Fischer, 2016). To examine the uncertainty from the reference period is out of the scope of this study. Rather, we tend to investigate the temporal change in the sensitivity of yield loss risk to drought severity under global warming. The RCP8.5 emission scenario is used to represent the upper bound of climate change impacts in the business-as-usual world.

## 3 Results

**Fig. 1:**
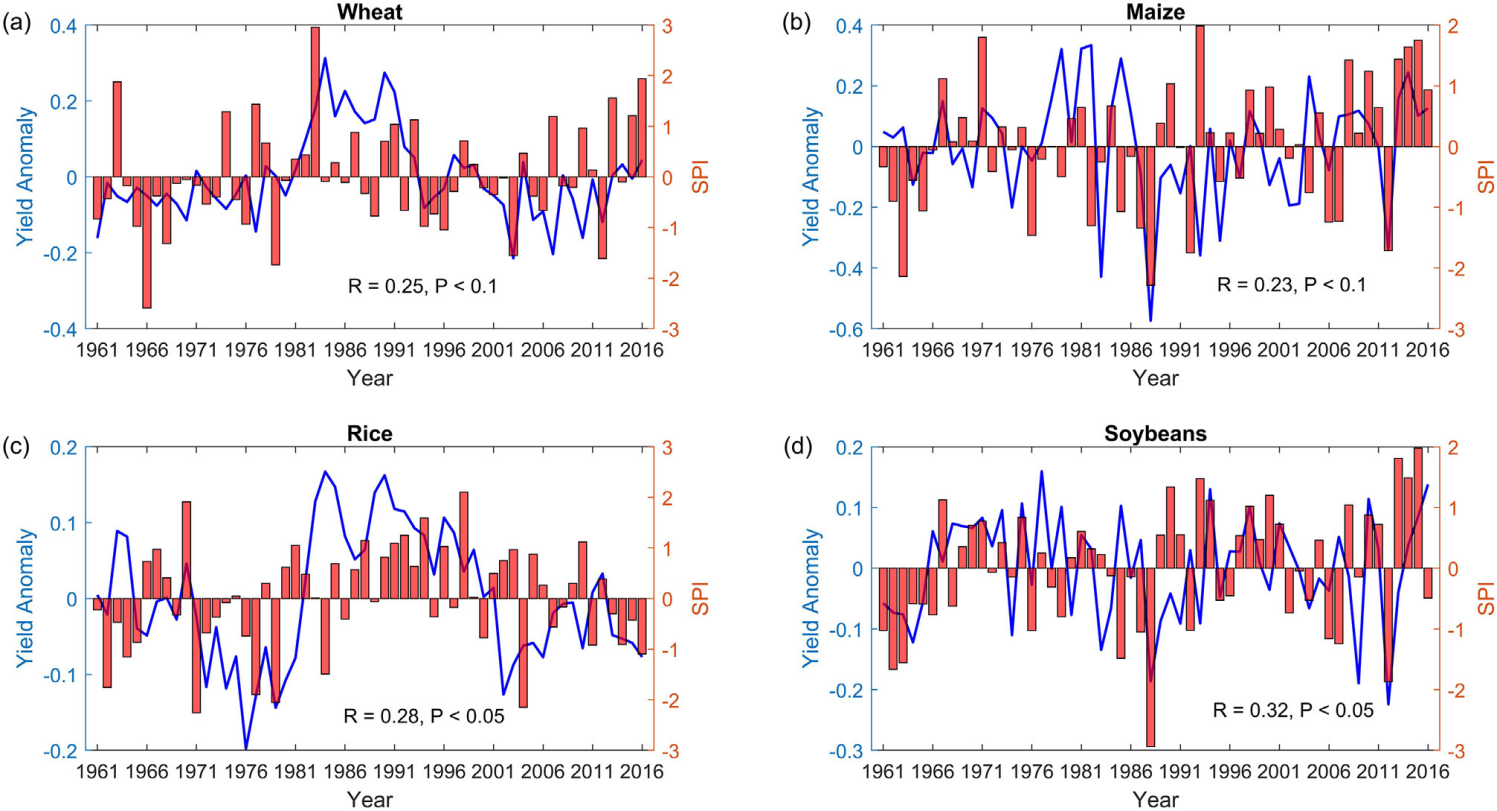
Temporal changes in de-trended annual yield anomaly (blue line) and growing season SPI (red bar) during 1961–2016 for global (a) wheat, (b) maize, (c) rice and (d) soybeans. The correlation coefficient (R) and statistical significance (*P*-value) are given.

Global agricultural productivity has exhibited substantial variations during the past decades, and crop yield reductions are often observed when dry conditions occurred (Fig. 1). Overall, crop yield variability can be explained by the drought index (i.e. SPI) for the study period. Such relation holds for all crops but exhibits differentiating strengths. Specifically, year-to-year variation of soybean shows the highest correlation with the drought index, followed by rice, wheat and maize. This is consistent with previous studies reporting larger impacts of precipitation on rice and soybeans than wheat and maize (Lobell and Field, 2007). A substantial decrease up to 25% in crop yield is observed in dry conditions (SPI < −0.8) as compared to wet conditions (SPI > 0.8). The negative impact of drought on crop yields is in line with previous studies at regional and global scales (Antwi-Agyei et al., 2012; Li et al., 2009; Lobell et al., 2014; Richter and Semenov, 2005; Troy et al., 2015; Zipper et al., 2016), although the magnitudes of estimations differ to certain extent due to difference in the time period and dataset used. Notably, a large portion of yield variation cannot be explained by the drought index, since several other factors influencing yield are not considered. Nevertheless, these results imply that occurrence of a drought event may not necessarily lead to a reduction of yield, confirming the importance of re-assessing the drought impacts in a probabilistic manner, as pursued in this study.

**Fig. 2:**
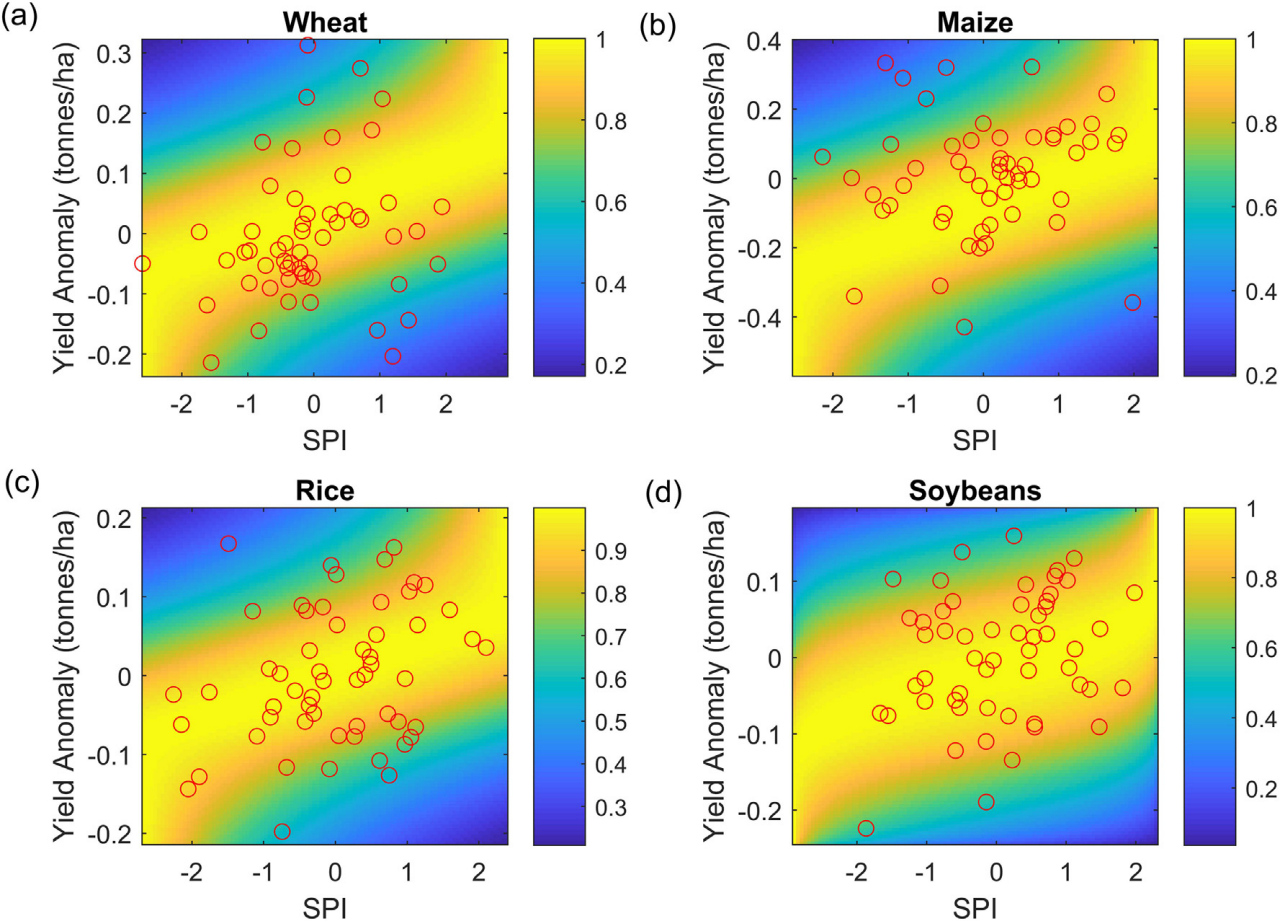
Joint distribution function fitted for global yield anomaly of (a) wheat, (b) maize, (c) rice and (d) soybeans and drought index (i.e. SPI). Each red circle denotes a pair of observed yield anomaly and SPI, while the background colors represent the probability densities. There are 56 red circles for the period 1961–2016 in each subplot. The specific copula model selected for each crop-region combination can be found in Table 5.

We then fit the joint distribution function between crop yields and SPI for the globe as a whole (Fig. 2). The Frank, Gumbel, Frank, and t models are selected for global wheat, maize, rice and soybeans, respectively, while the probabilistic model used for other country-crop combination is summarized in Table 5. The diverse model selections among crops and regions indicate that the relationship between yield anomaly and droughts depends on the region and crop of interest. Comparing the estimated probability distribution of yields with observations (red dots) shows that the majority of observed yields fall well within the high-density regions. Indeed, based on evaluation metrics including the maximum likelihood, Bayesian Information Criterion and Akaike Information Criteria (see Methods), the probabilistic model developed in this study is reliable for investigating crop yield responses to droughts of various severity.

**Table 5:** The best copula selected for each crop-region combination. The results for the top ten producing countries for each crop are shown.

Wheat		Maize		Rice		Soybeans	
Globe	Frank	Globe	Gumbel	Globe	Frank	Globe	t
China	Gumbel	United States	Clayton	China	Gaussian	United States	t
United States	Clayton	China	Gumbel	India	Clayton	Brazil	Clayton
India	Clayton	Brazil	Clayton	Indonesia	Gumbel	Argentina	Frank
Russian	Frank	Mexico	Gumbel	Bangladesh	Clayton	China	Gaussian
France	Clayton	Argentina	Clayton	Viet Nam	Clayton	India	Frank
Canada	Clayton	France	Clayton	Thailand	Gaussian	Paraguay	Frank
Ukraine	t	India	t	Myanmar	Frank	Canada	Clayton
Turkey	Frank	Ukraine	Gaussian	Japan	Clayton	Ukraine	Gumbel
Australia	Clayton	Romania	t	Philippines	Gumbel	Russian	Gumbel

**Fig. 3:**
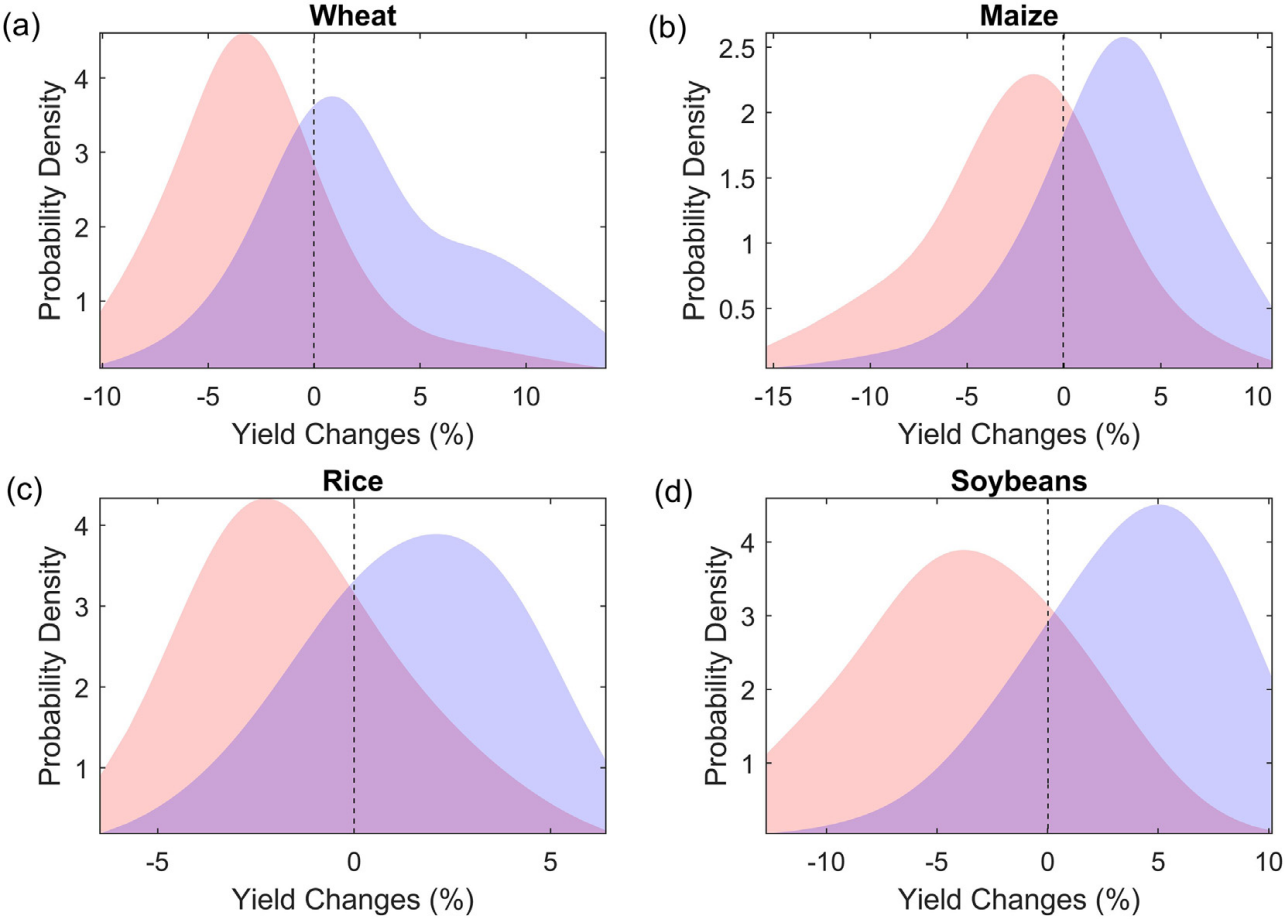
Conditional probability distribution of yield changes (%) relative to its long-term mean under moderate drought (red) and wet (blue) conditions for (a) wheat, (b) maize, (c) rice and (d) soybeans.

Based on the selected copulas, the conditional probability distributions of crop yields under dry and wet conditions are estimated (Fig. 3). A consistent increase of yield loss likelihood is observed for all crops from wet to dry conditions, as indicated by the shift of probability density curves to the left of long-term average. Under wet conditions, the probability of wheat yield loss (i.e. yield dropping below its long-term average as indicated by the vertical dashed line) for the globe as a whole is 41%, while the risk would increase by 17% to 58% when experiencing a moderate drought. Similar changes in yield distribution curves are found for maize, rice and soybean yields, exhibiting an increase in yield loss probability by 22%, 9% and 22%, respectively.

**Fig. 4:**
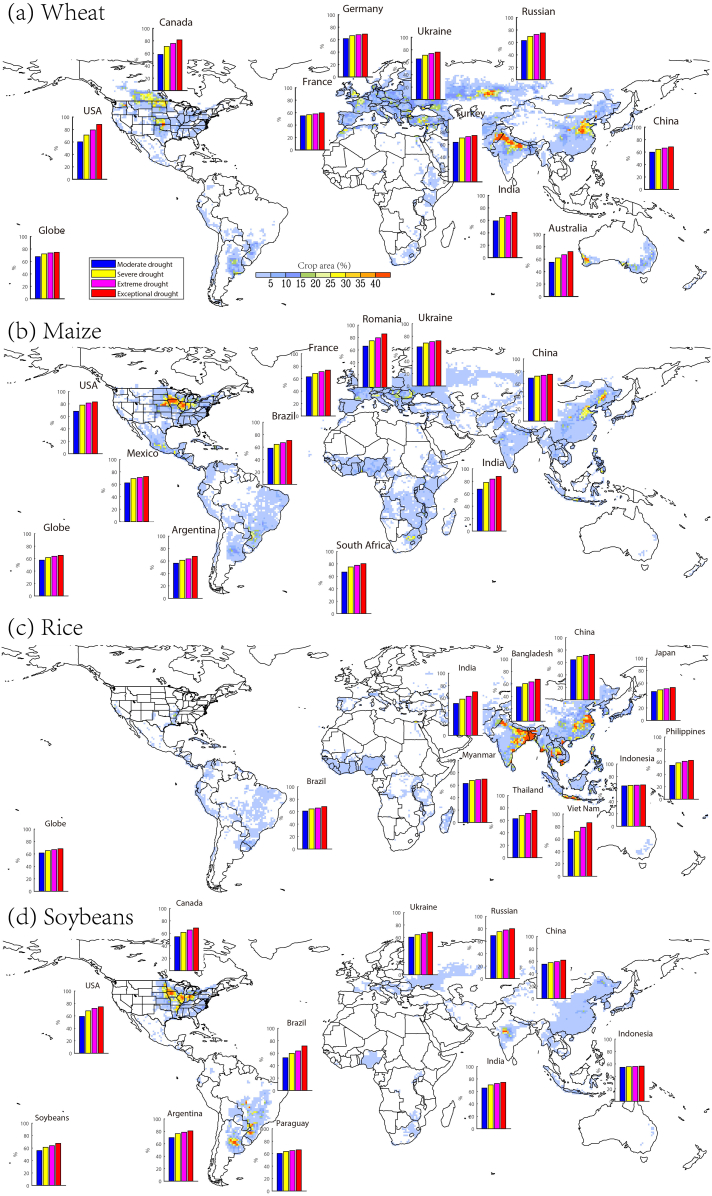
The probability (%) of yield loss (i.e. yield dropping below historical average) when experiencing a moderate (blue bar), extreme (yellow bar), severe (magenta bar) and exceptional drought (red bar) for (a) wheat, (b) maize, (c) rice and (d) soybeans. The top 10 producing countries for each crop are selected for illustration. The colored background map indicates the gridded crop area percentage (Portmann et al., 2010), based on which weights are assigned to gridded climate for spatial aggregations.

How will yield loss risk change with increase in drought intensity?. Here, the top 10 producing countries are selected for detailed analysis (Fig. 4), since they are highly relevant for global food market and security. Results for other small countries can be found in the Supplementary Tables. Globally, wheat is more vulnerable to droughts than maize, rice and soybeans, as indicated by the higher magnitude of yield loss probability under the four categories of droughts (i.e. moderate, severe, extreme and exceptional droughts). Regionally, the probability of wheat reduction is the highest in USA and Canada than other regions, and there is >80% probability that wheat reduction may fall below its long-term average when experiencing a drought at the exceptional category. As for maize, India shows the highest risk of yield reduction under droughts, while rice yield in Vietnam and Thailand are most vulnerable to droughts. Risk of drought-driven soybean yield reduction is the highest in USA, Russian and India, while relatively low risk is observed in other countries. The physical mechanisms behind the distinct spatial patterns are an open question since many factors could modulate drought impacts on farmers' fields. One obstacle has been lack of data on the physical and agronomical conditions that are relevant to crop growth and yield at fine scales. For example, agricultural management such as irrigation in US Great Plains can substantially modulate the response of maize yield to precipitation anomaly (Leng, 2017a). Nevertheless, the drought risk for crop yield in global major agricultural countries as revealed in this study provides valuable information for targeted adaptation and mitigation.

Comparing yield loss risk among moderate, extreme, severe and exceptional droughts can also give us valuable insights into the sensitivity of yield loss risk to the increase in drought severity. It is observed that with increase in drought severity, yield loss risk tends to grow progressively independent of crop types and regions. When drought severity shifts from moderate to exceptional, the USA and Canada would experience an increase of wheat loss risk by 20%, while Romania, USA and India show an increase in yield loss risk by 15%. India and Vietnam show the largest increase of rice reduction risk by 20% when experiencing a growth in drought severity from moderate to exceptional, while yield loss risk remains relatively stable in Indonesia in response to increase in drought severity. The likelihood of soybean reduction in Brazil would increase from 52% under a moderate drought to 71% under an exception drought. Importantly, the rate of risk growth tends to become lower with increase in drought severity. That is, the risk difference between severe and exceptional droughts is smaller than that between moderate and extreme droughts. This trend suggests that yield response to droughts is non-linear with drought severity, implying that yield loss risk under a drought category cannot be extrapolated for estimation under droughts of other categories. The non-linear response of yield loss risk to droughts implies that targeted mitigation and adaptation strategies should consider not only the crop type and region, but also the drought severity of interest.

**Fig. 5:**
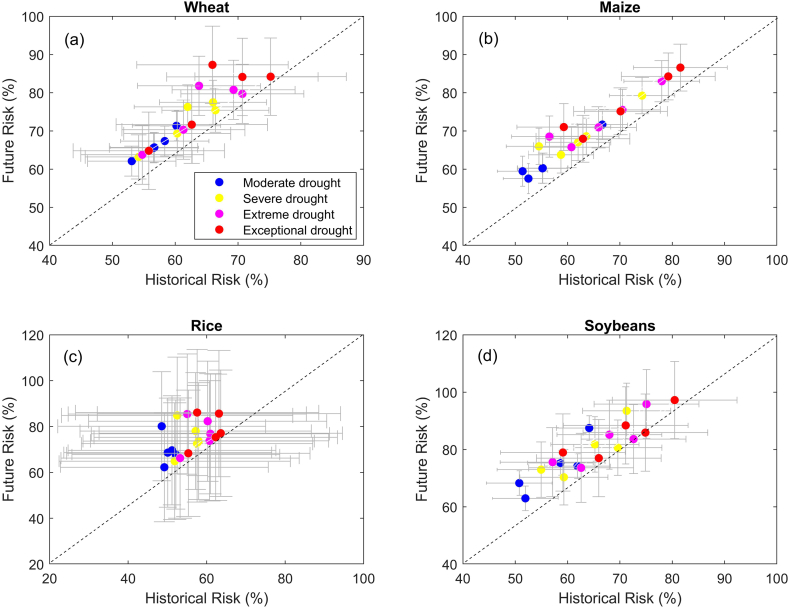
Projected changes in risk (%) of yield reduction in the future versus history as simulated by process-based crop models. Each dot represents the ensemble mean of risks simulated by 11 crop models under a given climate scenario, while the grey error lines indicate the corresponding uncertainties arising from crop models. The colors of dots represent the risk under various levels of drought severity. Here, five climate scenarios and four drought severity categories are considered, and there are 5 × 4 = 20 dots in each subplot.

How will the likelihood of drought-induced yield loss change in the future? Fig. 5 shows the projected yield loss risks under moderate, extreme, severe and exceptional droughts simulated by 11 state-of-the-art crop models driven by 5 climate models under RCP8.5 emission scenario. The multi-model ensemble mean gives estimates of yield loss risk that is comparable to those based on observations (Fig. 4) not only in the magnitude but also in the sensitivity of risk to drought severity, although considerable bias remains (Table 6). However, large discrepancy exists among crop models in simulating yield loss risk under droughts not only in the magnitude but also in terms of its sensitivity to increasing drought severity. This could be due to the differences in model structure, representation of environmental stress, agricultural management, and CO_2_ fertilization effect (Asseng et al., 2011; Elliott et al., 2015; Folberth et al., 2016; Rosenzweig et al., 2014). Such a validation suggests that it is reasonable to use the ensemble of process-based crop model simulations for assessing future changes in yield loss risk.

**Table 6:** Projected yield loss risk by the end of 21st century versus historical period for global wheat, maize, rice and soybeans.

Crop	History (%)	Future risk (%)	Difference (%)
Wheat	56.97	66.40	9.43
	61.78	72.33	10.55
	63.97	75.26	11.29
	66.05	78.41	12.36
Maize	56.21	61.82	5.61
	62.62	68.91	6.29
	66.34	72.74	6.40
	70.63	76.98	6.34
Rice	50.25	69.67	19.42
	55.42	74.77	19.35
	58.06	76.91	18.86
	60.40	78.49	18.10
Soybeans	57.48	73.63	16.16
	64.08	79.82	15.74
	67.09	82.75	15.66
	70.31	85.48	15.17

Projections based on the ensemble of crop models show that yield loss risk will increase in the future, with the magnitude depending on crop and severity of drought. The largest increase of yield loss risk is found for rice, followed by soybeans, wheat and maize. Notably, an uneven growth rate of yield loss risk is observed among the four drought categories. For example, larger risk of wheat yield loss is projected with increase in drought severity, while rice, maize and soybeans tend to show less sensitivity to increasing drought severity in the future (Fig. 5). Overall, drought-driven yield loss risk is projected to increase by 9%–12%, 5.6%–6.3%, 18.1%–19.4% and 15.1%–16.1 for wheat, maize, rice and soybeans, respectively, without considering adaptations or CO_2_ fertilization effect. The substantial increase of yield loss risk points to the need for effective adaptive measures for ensuring resilience in agricultural production in a warming climate with greater likelihood of more frequent and severe droughts (Dai, 2013; Huang et al., 2017; Sheffield and Wood, 2008).

## 4 Uncertainty and limitations

**Fig. 6:**
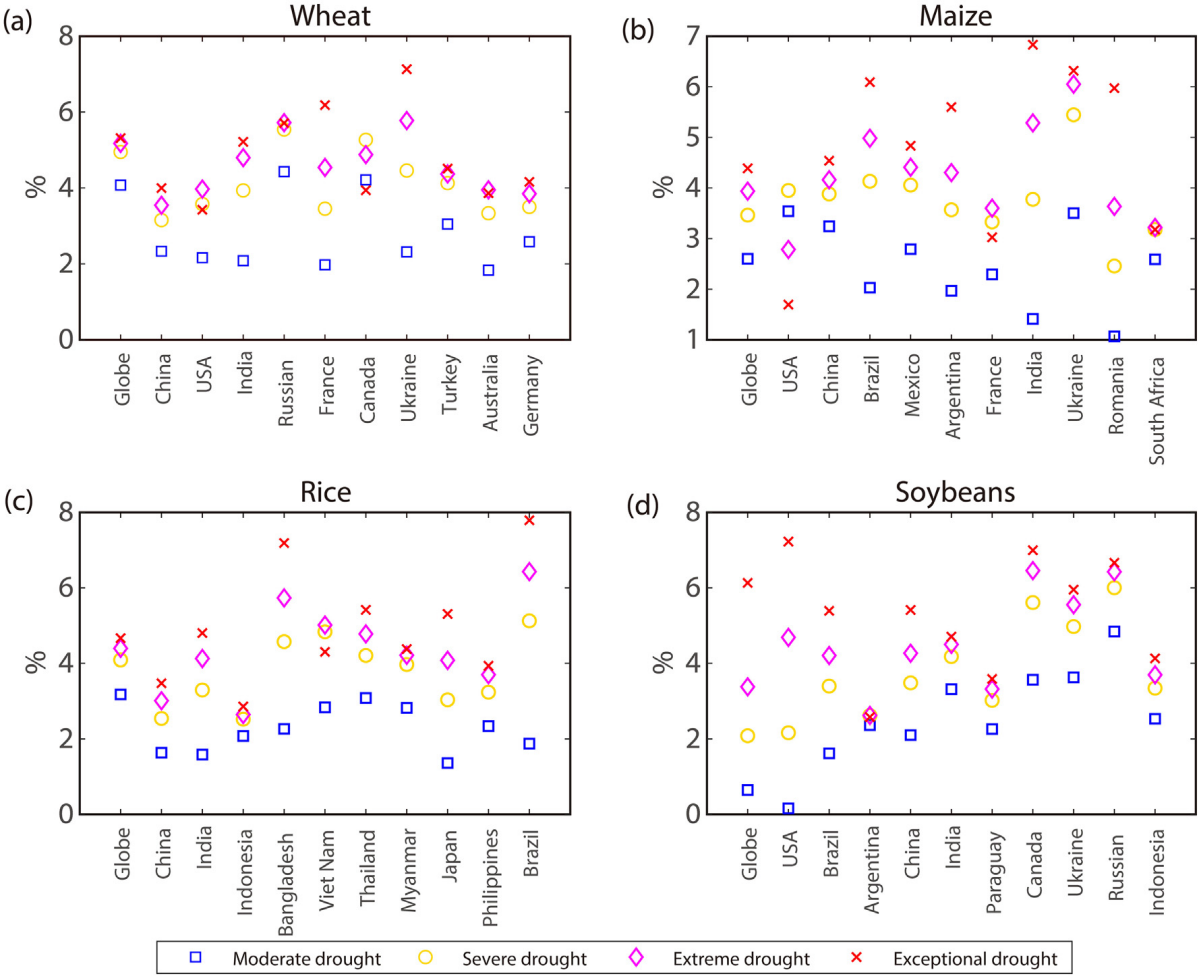
Uncertainties for the estimation of yield loss risk under droughts of various severity for each crop-region combination. The Markov chain Monte Carlo simulation technique is used within a Bayesian framework to derive the uncertainty based on the posterior distribution of copula parameters.

In this study, we develop a probabilistic model for estimating yield loss risk given an individual drought of specific severity. Serval uncertainty sources have to be acknowledged when interpreting the results of this study. For example, five popular copula models were selected to describe the dependency structure between yield and a drought index. Inherent uncertainty from the copula model itself would propagate and affect the estimation of yield loss risk under droughts. Here, we use the Markov chain Monte Carlo simulation technique within a Bayesian framework to infer the posterior distribution of copula parameters, based on which the uncertainty range arising from the copulas is estimated (see Methods). Fig. 6 shows the range between the lower and upper bounds of estimated yield loss risk under droughts for the four crops. Overall, the uncertainty is small compared to the estimated yield loss risk, with the magnitude depending on the crop-country combination of interest. Notably, the uncertainty magnitude tends to increase with drought severity, suggesting that estimation of yield loss risk under a moderate drought would be more reliable than those under more severe drought categories.

**Fig. 7:**
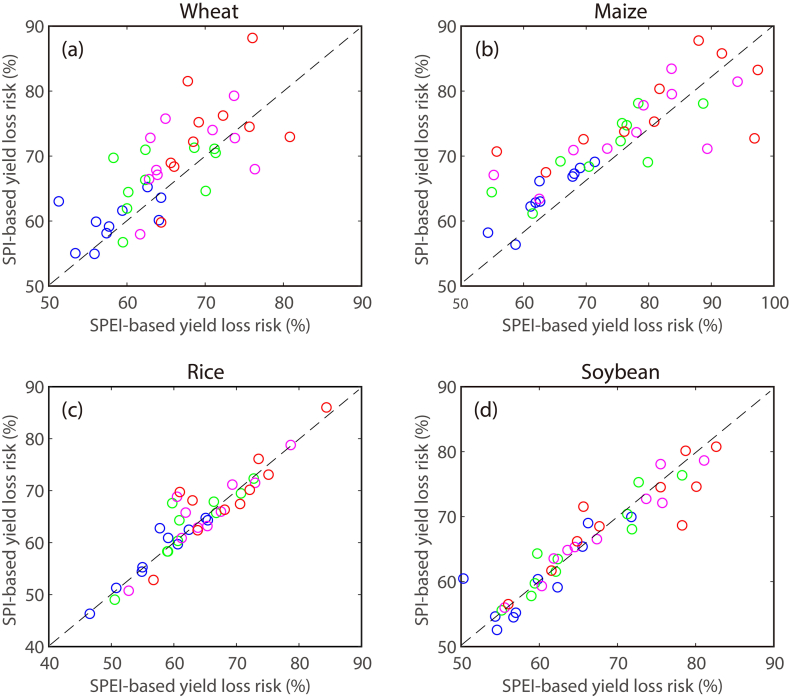
SPI-based yield loss risk versus *SPE*I-based yield loss risk for (a) wheat, (b) maize, (c) rice and (d) soybean. The top 10 producing countries are selected for illustration with each dot denoting a country, while the color represents the drought severity. The moderate, severe, extreme and exceptional droughts are indicated with blue, green, magenta and red, respectively.

It is well known that drought is driven by several other factors including temperature, which is also critical for crop growth and yield (Lobell et al., 2013; Lobell et al., 2014). Here, additional sensitivity analysis is conducted using a new drought index (i.e. *SPE*I) that accounts for the effects of temperature. As shown in Fig. 7, difference is observed between SPI- and *SPE*I-based yield loss risks. This difference suggests that drought-driven yield loss risk does depend on the definition of drought. Specifically, inclusion of temperature effects leads to considerable difference in the estimated yield loss risk of wheat and maize. However, the results remain largely similar for rice and soybean in response to SPI- and *SPE*I-based droughts. This could be attributed to the fact that rice and soybean are more sensitive to precipitation than temperature (Lobell and Field, 2007). Thus, consideration of temperature leads to minor change in the estimated yield loss risk under droughts. Besides climate factors, crop yields would be influenced by agricultural management such as irrigation (Leng et al., 2014; Müller et al., 2018) and fertilization (Leng et al., 2016; Müller et al., 2018; Stewart et al., 2005), conservation tillage (Karlen et al., 2013), multiple cropping (Seifert and Lobell, 2015), tile drainage (Schilling and Libra, 2003) and soil mulching (Qin et al., 2015). In addition, the CO_2_ fertilization effects tend to be beneficial for crop growth, but remain a large source of uncertainty (Schleussner et al., 2018). To which extent these compounding factors would reduce or amplify drought impacts on crop yield is not within the scope of this study, especially given the lack of detailed information and incomplete knowledge of the complex mechanisms behind yield variations.

We also acknowledge that the 11 process-based crop models and 5 climate models used in this study may not represent the full uncertainty range in the projection of future yield loss risk. In addition, future projections are conditional on the assumption of no adaptations, and thus may not be able to indicate the true future course of yield impacts. Therefore, future yield loss risks should not be interpreted as the correct magnitude of climate change impacts. Rather, the results are intended to provide a measure of the temporal evolution of yield loss risk under a warming climate, which has received little attention. Understanding the potential changes in the sensitivity of yield loss response to drought is a critical step towards planning and prioritizing effective adaptation options.

## 5 Summary and conclusion

This study provides a complementary method for assessing drought impact on global crop yields. Through developing a probabilistic model, we estimate yield loss risks (i.e. the probability of yield dropping below its long-term mean) under moderate, extreme, severe and exceptional droughts. Results show a significant association between droughts and yield reductions during the past decades. When experiencing an exceptional drought, the probability of yield loss could exceed 70% for soybean and maize, while the risk for wheat and rice is up to 68% and 64%, respectively. This prediction represents an increase of yield loss risk by 24%, 21%, 18% and 20% for soybean, maize, rice and wheat, respectively, when drought severity grows from moderate to the exceptional category. Notably, the rate of risk growth tends to become slower with increase in drought severity, suggesting the non-linear response of yields to droughts. Regionally, the risk of drought-driven wheat reduction is the highest in USA and Canada, where there is >80% probability that wheat reduction may fall below its long-term average given an exceptional drought. As for maize, India shows the highest risk of yield reduction, while rice yield in Vietnam and Thailand are most vulnerable to droughts. Risk of soybean yield reduction is the highest in USA, Russian and India, while relatively low risk is observed in other regions. Further analysis based on 11 process-based model simulations shows that yield loss risk will increase in the future, with the largest growth found for rice followed by soybeans, wheat and maize.

Enhancing the resilience of agricultural system to droughts would greatly benefit from improved understanding of the full range of possible outcomes of crop yields under a drought of specific severity. The insights abstained in this study enable more targeted efforts to manage drought risk, through adaptations of agricultural and water management practices, spatial diversification of crop production and the design of insurance instruments. The non-linear yield response to the increase in drought severity implies that future adaptations should be more targeted, considering not only the crop type and region but also the drought severity of interest.

## Appendix A Supplementary data

Supplementary tables: yield loss risk (%) under moderate, severe, extreme and exceptional droughts in each crop producing country of the world Image 1
