# Supplementary material for: Crop yield sensitivity of global major agricultural countries to droughts and the projected changes in the future
Source: Sci Total Environ. 2019 Mar 1;654:811–21. doi: 10.1016/j.scitotenv.2018.10.434 (PMC6341212; doi:10.1016/j.scitotenv.2018.10.434)
Supplement: Supplementary file 1 — Supplementary tables: yield loss risk (%) under moderate, severe, extreme and exceptional droughts in each crop producing country of the world [file mmc1.docx]

Crop yield sensitivity of global major agricultural countries to droughts and the projected changes in the future

Guoyong Leng and Jim Hall

Environmental Change Institute, University of Oxford, Oxford OX1 3QY, UK

Corresponding author address: Guoyong Leng, Environmental Change Institute, University of Oxford, Oxford OX1 3QY, UK

E-mail: [guoyong.leng@ouce.ox.ac.uk](mailto:guoyong.leng@ouce.ox.ac.uk)

**Supplementary Materials**

**Table S1** Wheat yield loss risk (%) under moderate, severe, extreme and exceptional droughts in each wheat producing country of the world

**Table S2** Same as Table S1 but for Maize

**Table S3** Same as Table S1 but for Rice

**Table S4** Same as Table S1 but for Soybean

Table S1 Wheat yield loss risk (%) under moderate, severe, extreme and exceptional droughts in each wheat producing country of the world.

| Country | % of global production | Moderate drought | Severe drought | Extreme drought | Exceptional drought |
| --- | --- | --- | --- | --- | --- |
| China | 15.318 | 59.892 | 64.452 | 66.442 | 68.374 |
| United States of America | 10.843 | 60.134 | 71.114 | 79.278 | 88.175 |
| India | 9.796 | 59.156 | 64.626 | 67.983 | 72.951 |
| Russian Federation | 9.071 | 63.028 | 69.725 | 72.801 | 75.208 |
| France | 5.362 | 54.950 | 56.736 | 57.961 | 59.787 |
| Canada | 4.475 | 58.128 | 70.969 | 75.757 | 81.524 |
| Ukraine | 3.629 | 65.231 | 71.282 | 74.008 | 76.230 |
| Turkey | 3.287 | 63.593 | 70.494 | 72.769 | 74.508 |
| Australia | 3.143 | 55.047 | 61.935 | 67.116 | 72.208 |
| Germany | 3.061 | 61.605 | 66.334 | 67.844 | 68.952 |
| Pakistan | 2.773 | 63.428 | 67.579 | 68.905 | 70.198 |
| Kazakhstan | 2.386 | 71.586 | 77.325 | 80.273 | 83.614 |
| U.K. of Great Britain and Northern Ireland | 2.103 | 51.432 | 53.558 | 55.192 | 57.868 |
| Argentina | 2.082 | 62.675 | 67.027 | 68.313 | 68.946 |
| Italy | 1.645 | 52.833 | 55.871 | 57.519 | 59.158 |
| Iran (Islamic Republic of) | 1.554 | 63.108 | 68.260 | 70.030 | 71.737 |
| Poland | 1.378 | 56.515 | 61.083 | 63.995 | 69.458 |
| Romania | 1.129 | 62.565 | 67.828 | 70.470 | 71.512 |
| Spain | 1.026 | 67.156 | 74.012 | 76.575 | 78.493 |
| Uzbekistan | 0.962 | 62.978 | 65.664 | 66.888 | 68.426 |
| Hungary | 0.882 | 53.310 | 54.589 | 55.386 | 56.213 |
| Egypt | 0.848 | 50.885 | 54.717 | 57.512 | 62.310 |
| Czech Republic | 0.823 | 55.824 | 59.490 | 60.798 | 62.091 |
| Bulgaria | 0.715 | 59.285 | 62.028 | 63.494 | 65.406 |
| Mexico | 0.623 | 55.786 | 58.662 | 60.198 | 61.963 |
| Brazil | 0.617 | 60.948 | 63.790 | 64.832 | 65.860 |
| Morocco | 0.590 | 62.387 | 68.683 | 71.026 | 74.126 |
| Afghanistan | 0.540 | 57.320 | 60.646 | 62.495 | 64.465 |
| Denmark | 0.537 | 60.080 | 63.223 | 64.819 | 67.082 |
| Serbia and Montenegro | 0.474 | 56.860 | 59.582 | 60.163 | 63.283 |
| Syrian Arab Republic | 0.472 | 64.886 | 78.474 | 85.930 | 92.430 |
| Ethiopia | 0.441 | 68.591 | 71.765 | 73.183 | 74.294 |
| Greece | 0.411 | 63.638 | 67.693 | 69.917 | 71.877 |
| South Africa | 0.360 | 55.625 | 59.111 | 60.252 | 61.381 |
| Belgium | 0.345 | 60.062 | 62.931 | 64.334 | 65.714 |
| Sweden | 0.332 | 57.460 | 60.582 | 61.963 | 64.083 |
| Lithuania | 0.326 | 57.900 | 62.218 | 64.929 | 69.336 |
| Slovakia | 0.325 | 61.334 | 75.341 | 82.929 | 92.101 |
| Algeria | 0.322 | 66.256 | 70.845 | 72.447 | 74.335 |
| Iraq | 0.287 | 62.700 | 68.287 | 71.513 | 75.939 |
| Turkmenistan | 0.286 | 69.722 | 76.385 | 79.547 | 82.756 |
| Belarus | 0.263 | 69.843 | 72.352 | 73.318 | 74.726 |
| Azerbaijan | 0.262 | 59.922 | 71.155 | 76.704 | 81.384 |
| Saudi Arabia | 0.259 | 51.201 | 56.561 | 62.364 | 72.101 |
| Chile | 0.259 | 56.999 | 61.119 | 62.664 | 64.182 |
| Austria | 0.248 | 45.566 | 47.936 | 48.879 | 55.012 |
| Tunisia | 0.196 | 59.028 | 61.369 | 62.551 | 63.980 |
| Netherlands | 0.187 | 55.597 | 59.891 | 62.897 | 68.512 |
| Moldova | 0.182 | 53.679 | 62.340 | 68.062 | 76.790 |
| Kyrgyzstan | 0.173 | 77.558 | 83.609 | 86.075 | 87.701 |
| Bangladesh | 0.163 | 62.171 | 66.573 | 68.683 | 71.038 |
| Nepal | 0.162 | 62.508 | 66.920 | 69.169 | 70.523 |
| Croatia | 0.158 | 50.221 | 53.467 | 55.748 | 57.441 |
| Latvia | 0.156 | 75.340 | 81.993 | 84.589 | 90.630 |
| Japan | 0.147 | 46.049 | 46.049 | 46.049 | 46.049 |
| Tajikistan | 0.115 | 65.482 | 73.976 | 78.188 | 82.828 |
| Uruguay | 0.107 | 47.750 | 48.258 | 48.443 | 48.628 |
| Finland | 0.105 | 51.417 | 55.375 | 58.463 | 63.699 |
| Ireland | 0.103 | 62.729 | 68.832 | 71.304 | 73.478 |
| Switzerland | 0.094 | 62.132 | 69.221 | 73.030 | 76.259 |
| Paraguay | 0.076 | 53.746 | 58.561 | 61.706 | 66.313 |
| Portugal | 0.074 | 52.548 | 54.010 | 54.736 | 55.706 |
| Albania | 0.068 | 53.712 | 55.554 | 56.386 | 57.050 |
| Mongolia | 0.064 | 81.982 | 89.992 | 91.859 | 93.399 |
| New Zealand | 0.062 | 55.731 | 57.193 | 57.839 | 58.483 |
| Sudan | 0.058 | 62.718 | 65.993 | 67.698 | 69.623 |
| The former Yugoslav Republic of Macedonia | 0.056 | 69.465 | 77.321 | 79.787 | 82.043 |
| Estonia | 0.051 | 50.728 | 58.138 | 63.858 | 71.025 |
| Bosnia and Herzegovina | 0.050 | 51.855 | 53.757 | 54.728 | 55.273 |
| Kenya | 0.048 | 53.823 | 54.914 | 55.458 | 55.784 |
| Armenia | 0.046 | 58.545 | 62.352 | 65.384 | 69.488 |
| Norway | 0.036 | 51.704 | 53.648 | 54.933 | 56.516 |
| Israel | 0.034 | 57.437 | 59.894 | 60.557 | 61.217 |
| Slovenia | 0.030 | 59.626 | 67.771 | 71.404 | 75.806 |
| Peru | 0.029 | 58.270 | 61.699 | 62.734 | 63.503 |
| Zimbabwe | 0.027 | 55.904 | 63.331 | 67.671 | 73.773 |
| Georgia | 0.026 | 60.328 | 72.330 | 79.330 | 88.789 |
| Yemen | 0.023 | 58.755 | 63.914 | 66.917 | 69.287 |
| Libyan Arab Jamahiriya | 0.023 | 68.046 | 71.119 | 72.775 | 74.504 |
| Dem People's Rep of Korea | 0.023 | 53.388 | 56.404 | 58.317 | 60.855 |
| Myanmar | 0.021 | 53.279 | 54.516 | 55.514 | 56.817 |
| Bolivia | 0.021 | 55.314 | 56.852 | 57.944 | 59.370 |
| Lebanon | 0.015 | 50.400 | 54.087 | 56.965 | 60.070 |
| United Republic of Tanzania | 0.014 | 57.985 | 62.130 | 64.675 | 68.411 |
| Jordan | 0.014 | 64.250 | 68.708 | 70.403 | 71.639 |
| Zambia | 0.012 | 60.187 | 65.463 | 68.119 | 70.776 |
| Republic of Korea | 0.012 | 58.868 | 61.129 | 62.366 | 63.345 |
| Colombia | 0.012 | 56.404 | 64.248 | 69.408 | 77.905 |
| Nigeria | 0.010 | 60.909 | 64.460 | 66.054 | 67.357 |
| Ecuador | 0.006 | 63.720 | 70.770 | 73.519 | 75.252 |
| Lesotho | 0.006 | 60.276 | 61.634 | 62.487 | 63.470 |
| Guatemala | 0.006 | 64.333 | 67.222 | 68.569 | 70.755 |
| Cyprus | 0.005 | 62.026 | 64.583 | 65.833 | 67.286 |
| Eritrea | 0.004 | 50.371 | 55.391 | 58.555 | 62.130 |
| Rwanda | 0.003 | 60.684 | 66.276 | 67.952 | 69.980 |
| Uganda | 0.002 | 53.349 | 59.105 | 62.906 | 68.420 |
| Angola | 0.002 | 59.780 | 66.582 | 70.526 | 76.046 |
| Bhutan | 0.002 | 56.567 | 59.127 | 60.136 | 61.135 |
| Mozambique | 0.001 | 64.045 | 68.189 | 69.712 | 70.900 |
| Mali | 0.001 | 65.466 | 72.887 | 76.529 | 80.163 |
| Burundi | 0.001 | 58.748 | 70.467 | 77.370 | 82.626 |
| Malta | 0.001 | 59.648 | 61.475 | 62.066 | 62.236 |
| Democratic Republic of the Congo | 0.001 | 57.559 | 60.875 | 62.663 | 65.047 |
| Namibia | 0.001 | 58.349 | 61.409 | 62.727 | 65.194 |
| Chad | 0.001 | 59.066 | 60.894 | 62.094 | 63.712 |

Table S2 Same as Table S1 but for Maize.

| Country | % of Total production | Moderate drought | Severe drought | Extreme drought | Exceptional drought |
| --- | --- | --- | --- | --- | --- |
| United States of America | 39.357 | 68.170 | 78.086 | 81.449 | 83.252 |
| China | 17.910 | 69.105 | 72.306 | 73.662 | 75.296 |
| Brazil | 5.806 | 58.211 | 64.445 | 67.085 | 70.696 |
| Mexico | 2.805 | 62.241 | 69.191 | 70.919 | 72.588 |
| Argentina | 2.457 | 56.371 | 61.160 | 63.411 | 67.499 |
| France | 2.127 | 63.003 | 68.372 | 71.150 | 73.765 |
| India | 2.018 | 67.290 | 78.133 | 83.439 | 87.775 |
| Ukraine | 1.974 | 62.845 | 69.053 | 71.127 | 72.726 |
| Romania | 1.672 | 66.141 | 74.737 | 79.532 | 85.793 |
| South Africa | 1.660 | 66.849 | 75.052 | 77.814 | 80.345 |
| Indonesia | 1.498 | 62.469 | 66.146 | 68.796 | 72.517 |
| Italy | 1.323 | 51.881 | 54.645 | 55.716 | 57.020 |
| Canada | 1.236 | 51.829 | 58.147 | 63.345 | 71.097 |
| Hungary | 1.143 | 60.763 | 68.892 | 72.810 | 79.291 |
| Serbia and Montenegro | 1.013 | 65.307 | 68.693 | 73.693 | 80.214 |
| Egypt | 0.868 | 57.412 | 59.027 | 59.655 | 60.661 |
| Russian Federation | 0.856 | 62.585 | 72.487 | 78.256 | 84.513 |
| Philippines | 0.769 | 63.086 | 67.424 | 68.939 | 70.417 |
| Nigeria | 0.767 | 60.261 | 68.937 | 73.612 | 78.271 |
| Ethiopia | 0.762 | 73.762 | 82.703 | 87.108 | 92.094 |
| Thailand | 0.636 | 61.538 | 74.147 | 80.871 | 89.259 |
| Spain | 0.548 | 60.330 | 66.976 | 71.122 | 76.831 |
| United Republic of Tanzania | 0.458 | 67.420 | 74.195 | 78.100 | 82.745 |
| Turkey | 0.440 | 57.933 | 66.872 | 71.885 | 79.462 |
| Kenya | 0.439 | 58.560 | 63.834 | 68.178 | 72.937 |
| Dem People's Rep of Korea | 0.409 | 65.271 | 69.566 | 72.092 | 75.072 |
| Germany | 0.390 | 61.103 | 67.954 | 70.597 | 75.130 |
| Bulgaria | 0.380 | 59.622 | 71.008 | 76.596 | 83.576 |
| Croatia | 0.350 | 53.955 | 66.334 | 74.337 | 82.311 |
| Pakistan | 0.330 | 54.897 | 57.904 | 59.535 | 62.145 |
| Malawi | 0.326 | 69.307 | 78.693 | 78.693 | 80.472 |
| Viet Nam | 0.315 | 57.178 | 61.457 | 64.326 | 68.052 |
| Greece | 0.292 | 64.158 | 67.986 | 70.325 | 72.906 |
| Zimbabwe | 0.270 | 72.339 | 80.301 | 82.532 | 84.557 |
| Austria | 0.263 | 55.100 | 58.345 | 59.504 | 61.403 |
| Moldova | 0.242 | 61.508 | 74.537 | 82.640 | 92.133 |
| Zambia | 0.241 | 58.849 | 63.800 | 66.624 | 68.481 |
| Nepal | 0.231 | 51.881 | 52.414 | 52.656 | 52.849 |
| Venezuela | 0.218 | 64.121 | 69.760 | 71.527 | 73.649 |
| Guatemala | 0.212 | 57.267 | 58.933 | 59.960 | 61.075 |
| Colombia | 0.208 | 52.645 | 55.089 | 56.128 | 56.816 |
| Paraguay | 0.184 | 58.126 | 59.054 | 59.474 | 60.032 |
| Uganda | 0.176 | 62.923 | 67.772 | 68.861 | 68.838 |
| Slovakia | 0.174 | 75.911 | 81.430 | 83.533 | 86.564 |
| Peru | 0.172 | 57.554 | 63.623 | 65.557 | 67.067 |
| Poland | 0.159 | 59.467 | 67.350 | 71.985 | 75.870 |
| Democratic Republic of the Congo | 0.158 | 62.491 | 65.043 | 65.795 | 66.292 |
| Ghana | 0.154 | 53.601 | 56.859 | 58.177 | 60.023 |
| Bosnia and Herzegovina | 0.146 | 58.875 | 74.244 | 81.909 | 89.577 |
| Mozambique | 0.143 | 51.952 | 57.447 | 61.895 | 68.211 |
| Cameroon | 0.141 | 56.980 | 59.198 | 60.133 | 60.836 |
| Chile | 0.141 | 55.077 | 58.595 | 60.462 | 62.594 |
| Portugal | 0.124 | 61.602 | 64.285 | 64.943 | 65.920 |
| Belgium | 0.123 | 76.647 | 88.313 | 93.167 | 97.938 |
| Iran (Islamic Republic of) | 0.119 | 59.322 | 62.880 | 64.265 | 65.624 |
| Benin | 0.104 | 55.093 | 57.153 | 58.013 | 59.111 |
| Czech Republic | 0.102 | 87.327 | 92.228 | 93.554 | 94.981 |
| El Salvador | 0.101 | 57.398 | 68.200 | 74.003 | 79.810 |
| Bolivia | 0.100 | 64.116 | 68.010 | 69.503 | 70.597 |
| Angola | 0.099 | 61.942 | 66.569 | 68.119 | 69.337 |
| Ecuador | 0.099 | 54.167 | 61.174 | 65.851 | 72.273 |
| Afghanistan | 0.098 | 56.432 | 63.755 | 67.884 | 75.201 |
| Honduras | 0.089 | 49.375 | 55.399 | 59.860 | 66.687 |
| Myanmar | 0.087 | 57.695 | 61.672 | 63.552 | 66.220 |
| Cote d'Ivoire | 0.084 | 59.211 | 65.565 | 70.170 | 75.085 |
| Mali | 0.084 | 68.781 | 74.580 | 76.769 | 78.818 |
| Burkina Faso | 0.078 | 58.896 | 67.255 | 73.328 | 80.258 |
| Kazakhstan | 0.076 | 62.129 | 73.534 | 82.226 | 85.908 |
| Kyrgyzstan | 0.074 | 66.180 | 76.941 | 82.389 | 89.435 |
| Georgia | 0.063 | 69.385 | 76.383 | 78.619 | 81.178 |
| Togo | 0.062 | 53.357 | 53.810 | 54.151 | 54.681 |
| Slovenia | 0.057 | 65.247 | 77.566 | 82.420 | 87.629 |
| Belarus | 0.056 | 89.252 | 96.194 | 98.384 | 98.384 |
| Nicaragua | 0.055 | 56.574 | 63.946 | 68.028 | 72.233 |
| Bangladesh | 0.055 | 51.377 | 53.117 | 53.784 | 54.317 |
| Lao People's Democratic Republic | 0.051 | 56.168 | 63.753 | 68.865 | 77.514 |
| Australia | 0.049 | 53.390 | 56.105 | 59.917 | 62.674 |
| Albania | 0.048 | 60.211 | 65.086 | 68.335 | 71.943 |
| Morocco | 0.048 | 63.092 | 68.807 | 70.752 | 72.160 |
| Uzbekistan | 0.046 | 56.099 | 61.684 | 64.943 | 69.504 |
| Guinea | 0.046 | 57.877 | 61.641 | 63.487 | 65.588 |
| Haiti | 0.042 | 56.177 | 59.551 | 60.935 | 62.030 |
| Cambodia | 0.040 | 54.891 | 57.511 | 58.454 | 59.390 |
| Uruguay | 0.038 | 58.042 | 60.954 | 61.909 | 62.620 |
| Madagascar | 0.038 | 59.130 | 65.007 | 68.747 | 70.313 |
| Cuba | 0.033 | 52.539 | 55.708 | 57.345 | 58.751 |
| Somalia | 0.030 | 50.371 | 53.451 | 56.320 | 61.184 |
| New Zealand | 0.028 | 53.230 | 58.478 | 61.529 | 66.306 |
| Iraq | 0.028 | 62.787 | 67.038 | 69.242 | 71.460 |
| Switzerland | 0.027 | 57.255 | 60.945 | 61.971 | 63.871 |
| The former Yugoslav Republic of Macedonia | 0.026 | 63.560 | 74.776 | 82.129 | 88.521 |
| Burundi | 0.026 | 58.316 | 61.588 | 62.846 | 64.326 |
| Rwanda | 0.025 | 63.677 | 71.314 | 76.250 | 82.352 |
| Senegal | 0.023 | 73.397 | 80.719 | 84.358 | 87.764 |
| Azerbaijan | 0.022 | 60.173 | 64.793 | 67.896 | 69.094 |
| Syrian Arab Republic | 0.022 | 63.553 | 67.158 | 68.737 | 69.971 |
| Tajikistan | 0.020 | 56.712 | 61.567 | 63.529 | 65.446 |
| Lesotho | 0.019 | 63.311 | 66.861 | 68.289 | 69.683 |
| Chad | 0.018 | 59.977 | 63.004 | 64.203 | 64.203 |
| Panama | 0.016 | 57.774 | 67.621 | 72.469 | 77.945 |
| Swaziland | 0.016 | 73.222 | 80.511 | 82.083 | 84.023 |
| Republic of Korea | 0.015 | 65.702 | 67.514 | 68.317 | 69.157 |
| Netherlands | 0.015 | 55.400 | 59.333 | 61.109 | 62.566 |
| Central African Republic | 0.014 | 59.236 | 62.061 | 63.068 | 63.816 |
| Bhutan | 0.012 | 49.757 | 49.841 | 49.880 | 49.906 |
| Timor-Leste | 0.012 | 60.594 | 67.690 | 71.837 | 75.649 |
| Denmark | 0.011 | 58.074 | 61.900 | 65.452 | 69.138 |
| Israel | 0.010 | 60.314 | 62.840 | 64.280 | 65.924 |
| Sri Lanka | 0.010 | 58.879 | 61.176 | 61.975 | 63.437 |
| Costa Rica | 0.010 | 50.437 | 52.762 | 54.276 | 56.778 |
| Yemen | 0.009 | 58.321 | 66.196 | 71.221 | 78.359 |
| Dominican Republic | 0.008 | 56.673 | 58.882 | 59.793 | 60.699 |
| Turkmenistan | 0.008 | 69.046 | 74.210 | 76.603 | 77.999 |
| Sudan | 0.007 | 59.832 | 62.611 | 64.664 | 67.204 |
| Malaysia | 0.007 | 57.074 | 60.490 | 62.715 | 65.414 |
| Namibia | 0.006 | 49.253 | 57.728 | 63.310 | 67.283 |
| Lithuania | 0.006 | 61.027 | 66.664 | 69.947 | 73.430 |
| Saudi Arabia | 0.005 | 70.380 | 75.843 | 78.039 | 80.088 |
| Belize | 0.005 | 58.631 | 67.414 | 73.304 | 80.723 |
| Gabon | 0.004 | 59.933 | 61.943 | 62.958 | 64.186 |
| Sierra Leone | 0.003 | 54.383 | 58.248 | 59.512 | 60.264 |
| Japan | 0.003 | 52.394 | 63.457 | 69.141 | 77.978 |
| Eritrea | 0.003 | 54.998 | 60.428 | 64.502 | 66.651 |
| Reunion | 0.002 | 54.723 | 59.429 | 62.186 | 65.783 |
| Armenia | 0.002 | 73.025 | 78.722 | 83.501 | 88.795 |
| Botswana | 0.002 | 57.075 | 63.383 | 67.613 | 72.914 |
| Oman | 0.002 | 65.702 | 67.514 | 68.317 | 69.157 |
| Guinea-Bissau | 0.002 | 63.553 | 67.158 | 68.737 | 69.971 |
| Congo | 0.002 | 61.813 | 66.175 | 67.848 | 69.074 |
| Cape Verde | 0.002 | 70.162 | 74.948 | 77.893 | 82.892 |
| Jordan | 0.001 | 64.908 | 70.949 | 74.791 | 78.035 |
| Niger | 0.001 | 73.222 | 80.511 | 82.083 | 84.023 |
| Mauritania | 0.001 | 64.761 | 72.089 | 74.732 | 75.985 |
| Kuwait | 0.001 | 53.269 | 55.828 | 57.729 | 61.707 |
| Comoros | 0.001 | 61.635 | 64.944 | 66.315 | 68.641 |
| Papua New Guinea | 0.001 | 60.464 | 61.606 | 61.965 | 62.595 |
| Jamaica | 0.001 | 62.152 | 63.998 | 64.872 | 65.967 |
| Lebanon | 0.001 | 59.074 | 61.348 | 63.147 | 65.121 |
| United Arab Emirates | 0.001 | 49.757 | 49.841 | 49.880 | 49.906 |
| Trinidad and Tobago | 0.001 | 55.492 | 61.025 | 64.220 | 71.038 |
| U.K. of Great Britain and Northern Ireland | 0.001 | 51.373 | 53.622 | 54.756 | 57.564 |
| Guyana | 0.001 | 63.851 | 66.135 | 66.912 | 67.253 |

Table S3 Same as Table S1 but for Rice

| Country | % of Total production | Moderate drought | Severe drought | Extreme drought | Exceptional drought |
| --- | --- | --- | --- | --- | --- |
| China | 32.395 | 64.304 | 69.476 | 71.511 | 73.082 |
| India | 20.984 | 51.290 | 58.312 | 62.886 | 70.165 |
| Indonesia | 8.314 | 64.742 | 65.726 | 65.971 | 66.336 |
| Bangladesh | 5.857 | 55.266 | 60.336 | 63.195 | 67.440 |
| Viet Nam | 4.601 | 59.695 | 72.327 | 78.784 | 86.011 |
| Thailand | 4.390 | 62.487 | 67.873 | 71.164 | 76.116 |
| Myanmar | 3.390 | 62.776 | 67.598 | 68.829 | 69.735 |
| Japan | 2.772 | 46.318 | 49.029 | 50.750 | 52.822 |
| Philippines | 2.050 | 54.422 | 58.311 | 60.877 | 62.382 |
| Brazil | 1.934 | 60.911 | 64.297 | 65.783 | 68.126 |
| United States of America | 1.427 | 53.815 | 60.017 | 64.363 | 69.560 |
| Republic of Korea | 1.337 | 67.416 | 73.142 | 74.901 | 76.168 |
| Pakistan | 1.161 | 58.103 | 62.407 | 63.801 | 65.171 |
| Egypt | 0.780 | 49.227 | 49.420 | 49.564 | 49.768 |
| Cambodia | 0.758 | 60.539 | 71.861 | 78.980 | 87.019 |
| Nepal | 0.657 | 52.871 | 55.295 | 56.840 | 59.246 |
| Madagascar | 0.524 | 60.803 | 63.652 | 65.111 | 66.959 |
| Dem People's Rep of Korea | 0.508 | 62.298 | 65.584 | 67.236 | 68.979 |
| Sri Lanka | 0.490 | 56.016 | 58.594 | 59.866 | 61.123 |
| Nigeria | 0.461 | 51.926 | 52.889 | 53.552 | 54.547 |
| Malaysia | 0.396 | 55.582 | 59.226 | 61.245 | 63.534 |
| Iran (Islamic Republic of) | 0.376 | 59.862 | 63.164 | 64.256 | 65.720 |
| Colombia | 0.346 | 58.768 | 64.863 | 69.140 | 74.441 |
| Lao People's Democratic Republic | 0.344 | 54.830 | 65.324 | 71.582 | 78.830 |
| Peru | 0.271 | 57.940 | 71.541 | 79.333 | 86.464 |
| Italy | 0.236 | 54.799 | 57.383 | 58.976 | 60.237 |
| Ecuador | 0.175 | 50.492 | 54.841 | 57.908 | 62.009 |
| Guinea | 0.173 | 48.962 | 50.983 | 51.656 | 52.159 |
| United Republic of Tanzania | 0.160 | 57.987 | 61.970 | 63.905 | 65.482 |
| Russian Federation | 0.144 | 55.060 | 63.983 | 70.069 | 79.590 |
| Argentina | 0.141 | 52.455 | 53.953 | 54.926 | 55.896 |
| Cote d'Ivoire | 0.134 | 61.295 | 65.450 | 67.471 | 69.391 |
| Uruguay | 0.131 | 59.055 | 63.811 | 65.716 | 66.835 |
| Mali | 0.130 | 74.101 | 81.077 | 82.712 | 84.234 |
| Australia | 0.129 | 52.152 | 59.126 | 63.032 | 67.429 |
| Venezuela | 0.118 | 51.429 | 53.322 | 54.150 | 55.333 |
| Sierra Leone | 0.118 | 58.229 | 61.234 | 62.307 | 63.103 |
| Spain | 0.117 | 59.558 | 64.933 | 67.214 | 69.102 |
| Dominican Republic | 0.095 | 63.764 | 69.412 | 71.937 | 74.224 |
| Cuba | 0.089 | 60.507 | 64.474 | 65.872 | 67.245 |
| Afghanistan | 0.083 | 58.355 | 60.924 | 61.931 | 63.115 |
| Turkey | 0.081 | 66.247 | 70.850 | 72.155 | 73.422 |
| Mexico | 0.079 | 58.419 | 61.028 | 62.485 | 64.049 |
| Guyana | 0.076 | 41.299 | 41.299 | 41.299 | 41.299 |
| Uzbekistan | 0.062 | 80.159 | 83.960 | 85.523 | 87.329 |
| Kazakhstan | 0.062 | 69.859 | 80.304 | 85.943 | 91.332 |
| Democratic Republic of the Congo | 0.055 | 67.447 | 74.551 | 77.039 | 78.919 |
| Bolivia | 0.045 | 64.655 | 67.614 | 68.691 | 69.399 |
| Senegal | 0.044 | 76.033 | 81.042 | 82.697 | 84.232 |
| Iraq | 0.044 | 49.481 | 52.570 | 54.694 | 57.404 |
| Panama | 0.044 | 65.672 | 73.307 | 76.231 | 81.291 |
| Liberia | 0.042 | 46.381 | 50.304 | 53.380 | 58.641 |
| Suriname | 0.041 | 58.450 | 62.509 | 64.298 | 65.699 |
| Nicaragua | 0.038 | 65.266 | 71.213 | 73.500 | 75.664 |
| Ghana | 0.038 | 57.455 | 63.392 | 67.141 | 73.088 |
| Costa Rica | 0.037 | 60.862 | 69.654 | 75.957 | 82.990 |
| Portugal | 0.031 | 55.632 | 63.359 | 67.728 | 74.251 |
| Paraguay | 0.028 | 57.490 | 64.561 | 69.452 | 75.949 |
| Greece | 0.028 | 50.358 | 54.199 | 60.146 | 66.094 |
| Chile | 0.024 | 50.013 | 53.474 | 55.201 | 56.430 |
| Haiti | 0.023 | 58.011 | 62.431 | 65.809 | 69.928 |
| Mozambique | 0.023 | 57.878 | 60.934 | 62.554 | 64.405 |
| Guinea-Bissau | 0.020 | 61.635 | 66.120 | 70.371 | 73.402 |
| Ukraine | 0.019 | 54.887 | 56.749 | 57.858 | 58.777 |
| Burkina Faso | 0.019 | 57.096 | 63.506 | 67.673 | 74.305 |
| France | 0.018 | 51.084 | 53.948 | 55.733 | 57.930 |
| Chad | 0.018 | 66.855 | 76.687 | 82.181 | 85.603 |
| Uganda | 0.016 | 68.816 | 71.740 | 73.287 | 75.017 |
| Turkmenistan | 0.015 | 57.930 | 64.658 | 68.963 | 73.249 |
| Cameroon | 0.014 | 61.449 | 68.731 | 74.425 | 79.450 |
| Malawi | 0.012 | 71.469 | 74.847 | 74.847 | 75.307 |
| Mauritania | 0.012 | 68.795 | 68.795 | 68.795 | 68.795 |
| Tajikistan | 0.011 | 67.865 | 74.813 | 78.068 | 79.790 |
| Bhutan | 0.011 | 51.024 | 55.580 | 58.983 | 63.484 |
| Togo | 0.011 | 57.743 | 59.999 | 61.082 | 61.986 |
| Niger | 0.010 | 56.900 | 63.611 | 68.516 | 74.495 |
| Benin | 0.010 | 62.557 | 64.841 | 66.158 | 68.272 |
| Romania | 0.010 | 65.952 | 72.222 | 75.614 | 78.210 |
| Ethiopia | 0.010 | 54.611 | 59.891 | 63.590 | 67.779 |
| Kenya | 0.010 | 52.975 | 56.945 | 58.892 | 60.173 |
| El Salvador | 0.009 | 62.016 | 72.317 | 77.788 | 83.207 |
| Timor-Leste | 0.009 | 61.266 | 72.397 | 82.486 | 94.494 |
| Bulgaria | 0.009 | 63.028 | 75.699 | 80.899 | 86.760 |
| Burundi | 0.007 | 50.373 | 52.685 | 53.795 | 56.197 |
| Honduras | 0.006 | 56.871 | 59.544 | 61.024 | 62.901 |
| Guatemala | 0.006 | 57.873 | 60.971 | 62.403 | 64.645 |
| Morocco | 0.006 | 54.514 | 58.607 | 61.208 | 63.541 |
| Hungary | 0.006 | 50.304 | 54.176 | 59.257 | 63.913 |
| Rwanda | 0.005 | 56.171 | 58.888 | 60.290 | 61.797 |
| The former Yugoslav Republic of Macedonia | 0.004 | 48.128 | 53.409 | 56.389 | 61.603 |
| Kyrgyzstan | 0.004 | 67.766 | 71.838 | 72.999 | 74.130 |
| Fiji | 0.004 | 55.822 | 57.177 | 57.787 | 58.746 |
| Central African Republic | 0.003 | 59.742 | 62.118 | 63.399 | 65.113 |
| Angola | 0.003 | 56.238 | 58.637 | 59.625 | 60.311 |
| Comoros | 0.003 | 49.562 | 51.066 | 51.842 | 52.951 |
| Zambia | 0.003 | 55.739 | 58.817 | 60.657 | 62.243 |
| Albania | 0.002 | 50.204 | 56.013 | 60.685 | 68.145 |
| French Guiana | 0.002 | 54.394 | 55.777 | 56.181 | 56.675 |
| Belize | 0.002 | 59.727 | 64.891 | 67.526 | 70.424 |
| Trinidad and Tobago | 0.002 | 64.587 | 68.483 | 70.866 | 73.944 |
| Somalia | 0.002 | 52.673 | 54.505 | 55.873 | 60.696 |
| Sudan | 0.002 | 62.342 | 66.396 | 68.277 | 70.250 |
| Azerbaijan | 0.002 | 62.805 | 80.029 | 88.406 | 94.277 |
| Hong Kong | 0.001 | 57.675 | 62.602 | 66.500 | 71.912 |
| Solomon islands | 0.001 | 55.356 | 56.385 | 56.385 | 56.513 |
| South Africa | 0.001 | 60.250 | 70.788 | 77.114 | 84.582 |
| Puerto Rico | 0.001 | 56.106 | 61.911 | 64.902 | 69.890 |
| Swaziland | 0.001 | 76.888 | 78.021 | 78.340 | 77.998 |
| Brunei Darussalam | 0.001 | 54.755 | 58.450 | 60.924 | 62.726 |
| Congo | 0.001 | 63.300 | 64.633 | 65.247 | 65.850 |

Table S4 Same as Table S1 but for Soybean

| Country | % of Total production | Moderate drought | Severe drought | Extreme drought | Exceptional drought |
| --- | --- | --- | --- | --- | --- |
| United States of America | 44.778 | 59.130 | 68.069 | 72.117 | 74.614 |
| Brazil | 21.347 | 52.570 | 59.735 | 63.561 | 71.540 |
| Argentina | 12.576 | 69.952 | 76.362 | 78.645 | 80.762 |
| China | 8.591 | 55.212 | 57.796 | 59.322 | 61.696 |
| India | 3.060 | 65.394 | 70.398 | 72.743 | 74.536 |
| Paraguay | 1.825 | 60.369 | 63.472 | 64.850 | 66.203 |
| Canada | 1.411 | 54.511 | 61.543 | 65.324 | 68.509 |
| Ukraine | 0.819 | 60.474 | 64.320 | 66.538 | 68.660 |
| Russian Federation | 0.714 | 69.011 | 75.296 | 78.083 | 80.136 |
| Indonesia | 0.659 | 54.630 | 55.582 | 56.004 | 56.531 |
| Bolivia | 0.625 | 60.138 | 66.608 | 70.968 | 74.982 |
| Italy | 0.375 | 54.788 | 58.337 | 60.054 | 62.592 |
| Uruguay | 0.302 | 50.845 | 54.970 | 57.239 | 61.835 |
| Mexico | 0.264 | 54.696 | 61.819 | 66.778 | 74.298 |
| Dem People's Rep of Korea | 0.256 | 54.695 | 57.924 | 59.932 | 61.898 |
| Nigeria | 0.191 | 66.719 | 71.375 | 74.071 | 76.101 |
| Japan | 0.158 | 56.592 | 65.076 | 70.788 | 75.816 |
| Thailand | 0.156 | 53.521 | 57.170 | 58.766 | 59.949 |
| Serbia and Montenegro | 0.146 | 61.692 | 65.724 | 66.496 | 68.032 |
| Republic of Korea | 0.145 | 57.711 | 61.295 | 62.465 | 63.334 |
| Romania | 0.140 | 82.533 | 91.157 | 93.406 | 94.790 |
| South Africa | 0.136 | 70.050 | 77.486 | 80.666 | 83.038 |
| France | 0.108 | 58.722 | 64.793 | 67.397 | 69.399 |
| Viet Nam | 0.080 | 58.227 | 67.088 | 70.922 | 75.891 |
| Croatia | 0.080 | 58.802 | 72.862 | 80.604 | 89.969 |
| Colombia | 0.072 | 52.864 | 54.539 | 55.738 | 57.217 |
| Iran (Islamic Republic of) | 0.070 | 64.810 | 70.230 | 72.181 | 73.683 |
| Malawi | 0.064 | 56.305 | 57.822 | 59.840 | 62.306 |
| Kazakhstan | 0.058 | 69.603 | 77.911 | 81.361 | 84.354 |
| Myanmar | 0.054 | 63.653 | 66.549 | 68.168 | 69.892 |
| Bangladesh | 0.053 | 61.917 | 65.288 | 67.997 | 69.182 |
| Zimbabwe | 0.052 | 64.297 | 72.115 | 74.457 | 76.657 |
| Austria | 0.050 | 70.598 | 77.443 | 80.082 | 81.898 |
| Turkey | 0.047 | 57.723 | 61.908 | 63.872 | 65.945 |
| Egypt | 0.046 | 57.176 | 60.137 | 61.502 | 63.407 |
| Ecuador | 0.042 | 58.272 | 64.930 | 67.957 | 72.591 |
| Australia | 0.042 | 58.689 | 63.449 | 65.091 | 66.059 |
| Uganda | 0.040 | 49.039 | 51.108 | 52.984 | 55.369 |
| Hungary | 0.038 | 60.783 | 67.592 | 72.598 | 79.008 |
| Zambia | 0.035 | 56.995 | 65.676 | 70.738 | 78.895 |
| Cambodia | 0.034 | 60.072 | 62.387 | 63.703 | 64.786 |
| Moldova | 0.029 | 63.657 | 74.179 | 78.936 | 83.002 |
| Guatemala | 0.025 | 51.597 | 55.311 | 57.495 | 60.865 |
| Bulgaria | 0.020 | 72.606 | 79.913 | 84.226 | 88.410 |
| Benin | 0.020 | 44.301 | 54.606 | 61.778 | 72.088 |
| Slovakia | 0.017 | 66.541 | 69.722 | 71.940 | 74.364 |
| Ethiopia | 0.016 | 59.680 | 66.954 | 71.143 | 75.171 |
| Venezuela | 0.011 | 63.091 | 69.119 | 73.025 | 78.530 |
| Rwanda | 0.011 | 59.720 | 65.337 | 67.596 | 70.425 |
| Nepal | 0.009 | 56.022 | 60.633 | 64.044 | 68.500 |
| Czech Republic | 0.008 | 60.100 | 65.358 | 69.059 | 75.540 |
| Democratic Republic of the Congo | 0.008 | 63.543 | 66.997 | 68.115 | 68.667 |
| Spain | 0.006 | 51.948 | 56.722 | 59.485 | 62.712 |
| Nicaragua | 0.006 | 57.979 | 63.866 | 66.452 | 68.531 |
| Bosnia and Herzegovina | 0.006 | 58.417 | 69.293 | 77.157 | 84.595 |
| Burkina Faso | 0.006 | 67.763 | 69.796 | 70.925 | 72.282 |
| Angola | 0.005 | 66.110 | 68.834 | 70.204 | 71.649 |
| Cameroon | 0.005 | 47.510 | 52.919 | 56.729 | 62.592 |
| Lao People's Democratic Republic | 0.005 | 52.853 | 58.478 | 62.925 | 70.804 |
| Germany | 0.004 | 51.184 | 56.723 | 60.635 | 63.965 |
| Syrian Arab Republic | 0.004 | 74.916 | 89.034 | 93.362 | 94.740 |
| Switzerland | 0.003 | 66.323 | 69.912 | 72.092 | 74.936 |
| Greece | 0.002 | 50.571 | 56.131 | 59.657 | 64.577 |
| Philippines | 0.002 | 50.757 | 57.250 | 62.503 | 67.370 |
| Peru | 0.002 | 54.897 | 56.756 | 57.820 | 59.130 |
| Albania | 0.002 | 63.894 | 70.915 | 74.542 | 79.966 |
| Gabon | 0.002 | 41.743 | 50.770 | 58.028 | 69.199 |
| Honduras | 0.002 | 57.891 | 70.797 | 78.043 | 86.980 |
| Sri Lanka | 0.002 | 59.145 | 61.287 | 62.171 | 63.335 |
| El Salvador | 0.002 | 62.388 | 67.000 | 69.352 | 73.558 |
| Mali | 0.002 | 68.908 | 68.908 | 68.908 | 68.908 |
| Kenya | 0.002 | 62.458 | 64.697 | 64.697 | 65.331 |
| Morocco | 0.002 | 79.240 | 84.869 | 86.691 | 87.697 |
| Liberia | 0.002 | 54.150 | 56.605 | 58.055 | 60.655 |
| United Republic of Tanzania | 0.002 | 64.285 | 70.764 | 74.177 | 76.999 |
| Georgia | 0.001 | 72.554 | 76.050 | 77.277 | 78.069 |
| Poland | 0.001 | 58.693 | 66.258 | 72.702 | 80.381 |
| Cote d'Ivoire | 0.001 | 63.801 | 66.637 | 68.208 | 69.613 |
| Pakistan | 0.001 | 69.106 | 72.767 | 74.711 | 77.368 |
| Chile | 0.001 | 66.076 | 69.672 | 73.069 | 75.740 |
| Belize | 0.001 | 66.361 | 77.670 | 83.090 | 89.143 |
| Burundi | 0.001 | 64.434 | 74.148 | 79.901 | 86.614 |
| Togo | 0.001 | 58.486 | 69.617 | 76.702 | 83.086 |
| Timor-Leste | 0.001 | 71.143 | 76.728 | 81.883 | 89.766 |
| Iraq | 0.001 | 41.539 | 48.461 | 57.025 | 65.335 |
| Bhutan | 0.001 | 62.941 | 65.294 | 66.475 | 68.304 |
| Costa Rica | 0.001 | 60.430 | 62.252 | 64.014 | 67.087 |
| Slovenia | 0.001 | 54.074 | 58.699 | 61.531 | 65.326 |
